# Deep learning and generative artificial intelligence in aging research and healthy longevity medicine

**DOI:** 10.18632/aging.206190

**Published:** 2025-01-16

**Authors:** Dominika Wilczok

**Affiliations:** 1 Duke University, Durham, NC 27708, USA; 2 Duke Kunshan University, Kunshan, Jiangsu 215316, China

**Keywords:** aging, generative artificial intelligence, deep learning, deep aging clocks, healthy longevity medicine

## Abstract

With the global population aging at an unprecedented rate, there is a need to extend healthy productive life span. This review examines how Deep Learning (DL) and Generative Artificial Intelligence (GenAI) are used in biomarker discovery, deep aging clock development, geroprotector identification and generation of dual-purpose therapeutics targeting aging and disease. The paper explores the emergence of multimodal, multitasking research systems highlighting promising future directions for GenAI in human and animal aging research, as well as clinical application in healthy longevity medicine.

## INTRODUCTION

The term “Artificial Intelligence” (AI) was coined at the seminal Dartmouth conference in 1956 [1]. While there are multiple definitions of AI, the United States National Artificial Intelligence Act of 2020 defines it as a machine-based system that can, for a given set of human-defined objectives, make predictions, recommendations or decisions influencing real or virtual environments [2].

**Figure 1:**
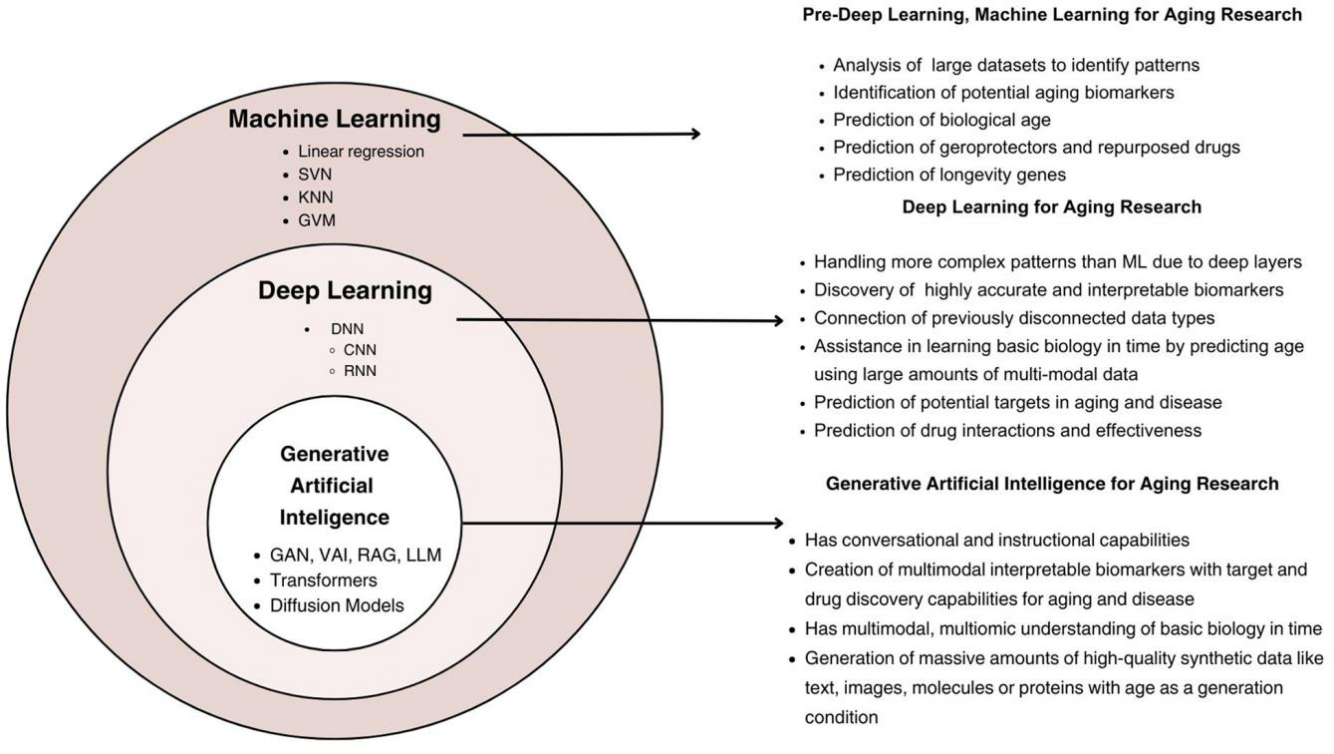
**The layered structure of machine learning, deep learning, and generative artificial intelligence in the context of aging research.** ML encompasses foundational methods, including linear regression and support vector machines, for biomarker identification and biological age prediction. DL builds on ML, employing architectures such as convolutional and recurrent neural networks to analyze complex, multimodal datasets. GenAI extends DL capabilities through generative models, including GANs and transformers, enabling synthetic data generation, multimodal biomarker creation, and advanced applications in drug discovery and aging-related interventions.

AI encompasses various subfields, including Machine Learning (ML) and DL. Figure 1 illustrates the hierarchical relationship between the different types of AI and their functionality and capabilities. ML refers to algorithms and statistical models that enable computers to perform tasks without explicit instructions, relying on patterns and inference [3]. DL, a subset of ML, uses neural networks with many layers (hence “deep”) to analyze various types of data [4]. DL was first applied to aging research in 2015-2016 with the publication of deep aging clocks (DACs) by A. Zhavoronkov’s group [5]. Since then, many techniques in DL, such as Generative Adversarial Networks (GAN), Large Language Models (LLM), and Denoising Diffusion Probabilistic Models, have been applied to advance aging research, and for comprehensive health assessment in healthy longevity medicine (Figure 1). The results of these first applications were presented in workshops and conference presentations before they were published, such as using DL to automate multispecies phenotyping in aging research [6], DL for drug clustering and automatic cell staining as well as for generating synthetic data with age as generation condition [7] or screening the small molecule library for DNA-repairing compounds [8].

Several recent reviews have examined the impact of AI in aging research, but none have specifically focused on GenAI and DL. For instance, Marino et al. (2023) reviewed the application of AI to aging research within specific hallmarks of aging [9]. Czaja and Ceruso (2022) concentrated on how AI integration into daily activities, such as continuous health monitoring, can enhance the quality of life for older adults [10]. Similarly, Bernal et al. (2024) conducted a systematic literature review focusing on the application of AI to age-related sociodemographic, cognitive and physical changes, without extensive focus on research or clinical applications [11]. Meng et al. (2024) narrowed their scope to biological age estimation [12], and Wang et al. (2023) discussed biomarkers and aging clocks [13]; however, neither provided a comprehensive analysis centered on GenAI and DL. Steurer et al. (2024) offered an overview of multimodal transformers—a type of GenAI—in aging research [14], while Lyu et al. (2024) extended the scope of their publication vastly beyond DL and GenAI [15]. Although Zhavoronkov et al. (2019) presented an in-depth analysis of DL in aging research [16], the rapid advancements in AI techniques since then necessitate an updated review. Addressing this gap, the present review encompasses 125 peer-reviewed studies on the application of DL and GenAI in aging research and healthy longevity medicine, covering works from 2016 to 2024.

## Overview of deep learning methods used in aging research

### Deep learning

DL significantly advanced AI by enabling computers to learn from vast, complex datasets [17]. Unlike many traditional ML approaches, which can include a variety of methods such as decision trees and support vector machines, DL distinguishes itself by using Deep Neural Networks (DNNs) with multiple layers, capable of automatically discerning and interpreting intricate patterns across diverse, previously disconnected data types [18]. In aging research, they were found to be particularly valuable due to their ability to analyze diverse longitudinal data types, such genomic sequencing, blood biomarker trends, and daily physical activity logs, allowing researchers to study and interpret patterns in the aging process [16, 19].

Within the broader category of DNNs, specific architectures have been developed to tackle different types of data and tasks. Among others, Convolutional Neural Networks (CNNs) identify patterns in grid-like data, such as images, by capturing spatial features across multiple layers [20]. They are optimized for processing visual information and streamline image analysis. In contrast, Recurrent Neural Networks (RNNs) capture patterns in sequential data as such as genomic sequences, speech, and real-time sensor data by using feedback loops that allow information to be retained across steps in a sequence. They incorporate information from previous inputs to influence future outputs, using a primitive form of memory [21].

DL is the foundation for the development of more advanced AI algorithms, particularly generative models. These models not only analyze existing data, but can create new, synthetic data, which has significant implications for fields requiring complex data generation or augmentation.

### Generative artificial intelligence

**Figure 2:**
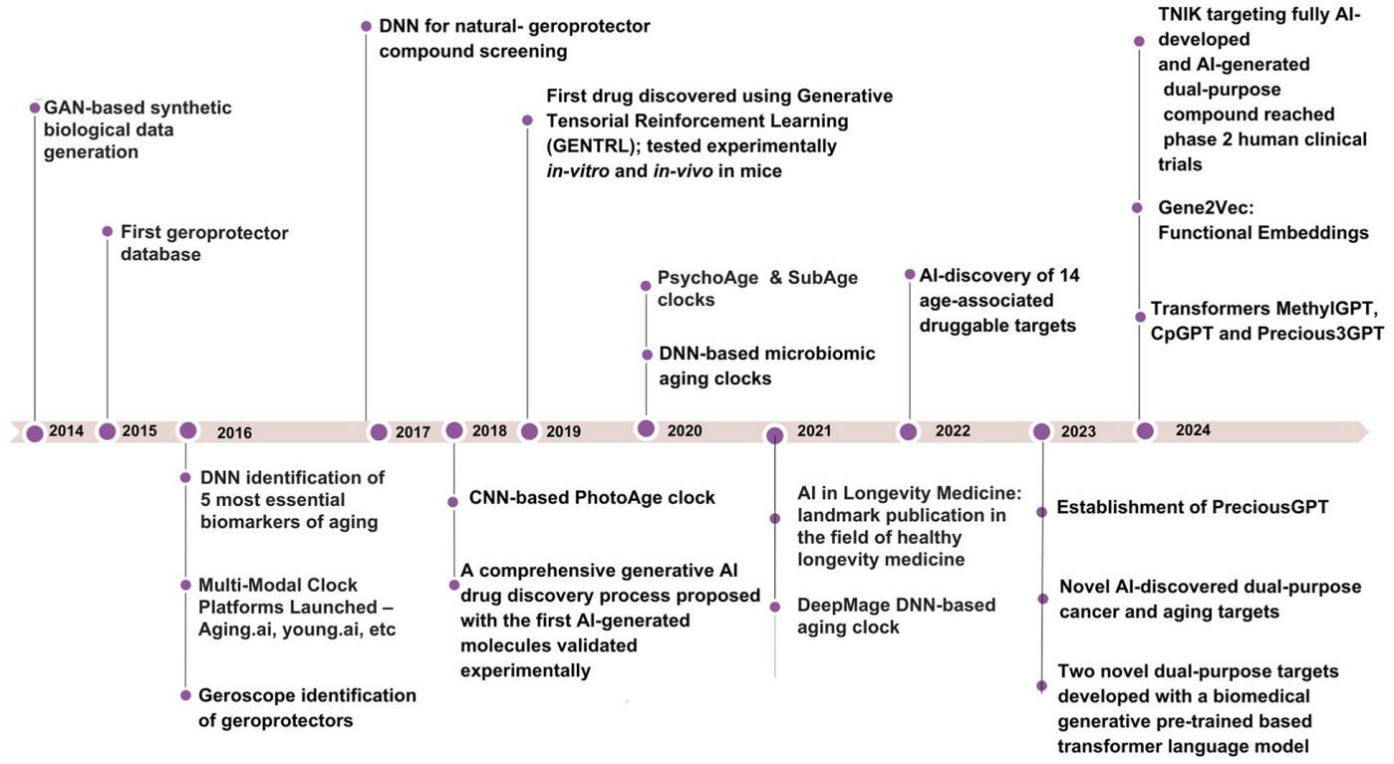
A timeline of major milestones in AI applications for aging research from 2014 to 2024.

GenAI algorithms significantly expand the capabilities of ML by producing diverse outputs, including text, images, and simulations. These algorithms leverage large-scale datasets to generate results tailored to specific parameters and requirements [22]. While many GenAI systems employ advanced neural network architectures, the field encompasses a variety of techniques for creating new data based on learned patterns and distributions. A prominent example of GenAI is the Generative Adversarial Networks (GANs), introduced by Goodfellow et al. in 2014 [23, 24]. GANs have demonstrated effectiveness in various medical applications, including medical image analysis [25–28] and disease progression modeling [29, 30]. In the context of aging research, GenAI techniques like GANs have shown promise in several areas. They have facilitated the development of biomarkers and early geroprotective interventions [31, 32], advanced understanding of age-related changes, and improved dataset balance for training more accurate predictive models. For instance, Campello et al. (2022) used GANs to synthesize aged and rejuvenated cardiac images from cross-sectional data, modeling realistic age-related changes in the heart [33]. Additionally, GenAI has been applied to protein function prediction [34] and the generation of synthetic biological data for hypothesis testing and model validation [35]. Figure 2 (adapted from Zhavoronkov et al., 2021, [36]), overviews the use of GenAI in various areas of aging research.

While GANs have been instrumental in advancing GenAI, recent years have seen the emergence of new architectures offering increased stability and quality of generated samples. GENTRL (Generative Tensorial Reinforcement Learning) combines GANs, Reinforcement Learning, and Deep Tensor Neural Networks to design novel small molecules with specified properties [37]. It was applied to identify inhibitors targeting fibrosis, a common consequence of the interplay of multiple hallmarks of aging [38, 39], exemplifying how GenAI in drug discovery can contribute to developing dual-purpose therapeutics for aging and diseases.

Transformers, popularized by Vaswani et al. (2017) [40], have revolutionized natural language processing through their self-attention mechanisms. These models excel in processing sequential data by capturing long-range dependencies, overcoming limitations of previous architectures [41]. While initially developed for language tasks, transformers’ versatility has led to their adaptation for various GenAI applications, including image generation and the creation of synthetic biological sequences [42].

Multimodal transformers, such as Google’s Gato [43] and *Insilico* Medicine’s PreciousGPT models [44, 45], allow for generalist multi-tasking. These models are trained on various data types to perform multiple tasks and go beyond traditional transformer-based synthetic data generation.

**Figure 3:**
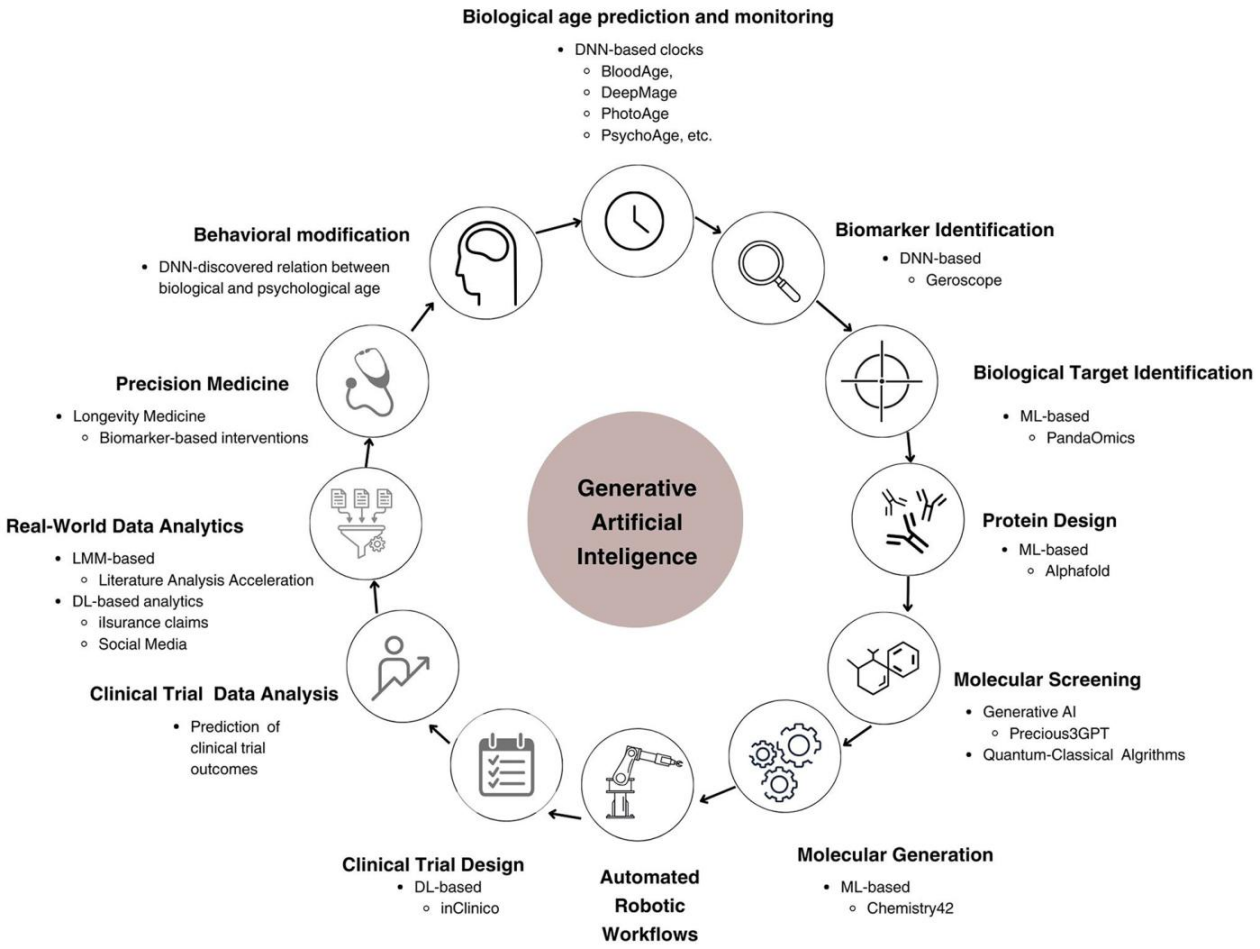
Diverse applications of GenAI across aging research.

Built using transformer architecture, LLMs have emerged as a significant part of natural language processing [46], LLMs use vast sets of textual data to learn linguistic patterns, syntax, and semantics enabling them to perform a wide range of language-based tasks. Retrieval Augmented Generation improves LLMs by combining traditional generative capabilities with a two-component retrieval mechanism system, fetching and integrating relevant documents [47]. It allows LLMs to access and incorporate external information in real-time, significantly improving response accuracy and contextual relevance, making natural language processing more reliable. In aging research, LLMs have shown potential for analyzing and summarizing vast amounts of scientific literature, potentially accelerating the discovery of aging-related insights and drug targets [48, 49]. Figure 3 demonstrates the timeline of DL and GenAI application to aging research.

Recent advancements have integrated transformer-inspired techniques across diverse domains, including protein structure prediction. DeepMind incorporated attention mechanisms into their AlphaFold models, significantly improving prediction accuracy. AlphaFold 3 utilizes a diffusion-based architecture and a “pairformer” module to focus on relevant parts of the input protein sequence [50]. This approach allows the model to capture complex dependencies and spatial relationships between distant amino acids, much like transformers managing word relationships in sentences, predicting the protein’s 3D structure with remarkable accuracy. It has significant implications for aging research, potentially accelerating the discovery of age-related protein interactions and therapeutic targets [51].

Another significant advancement in GenAI is the development of Denoising Diffusion Probabilistic Models (DDPMs). Introduced by Ho et al. (2020) [52], DDPMs have rapidly gained popularity for their effectiveness in image generation. These models work by gradually denoising a random noise distribution to produce high-quality synthetic images, demonstrating a novel approach to handling the complexities of image synthesis. The integration of DDPMs with transformer-based architectures enhances their performance, enabling more stable and robust generation of synthetic data across various domains, including text, images, and biological sequences [53].

Diffusion-based regression neural networks are a new type of DNN designed to improve regression tasks by leveraging diffusion processes [54]. These models iteratively transform raw input features into more meaningful representations, enhancing predictive accuracy.

## Recent AI applications in aging biomarker studies

### DL in biomarker of aging development

Biomarkers of aging are quantitative parameters measured in an organism to estimate biological age [55]. They may also reflect mortality risk, frailty, and other age-associated conditions. There are three fundamental categories of biomarkers of aging: molecular (multiomics, or other laboratory parameters), physiological (measured by functional performance) and digital (obtained through digital devices) [55]. To support their development and validation, biomarker consortiums have been established in the United States [55] and China [56]. These consortiums focus on defining the critical attributes biomarkers must possess, such as responsiveness to interventions, while also providing comprehensive roadmaps to facilitate their translation and validation [57].

In 2022, a group at the University of Copenhagen led by M. Scheibye-Knudsen published a DL approach to establishing a deep biomarker of cellular senescence [58]. Their CNN model trained on nuclear morphology high-content microscopy images achieved up to 95% accuracy in detecting senescence. Training the custom CNN precursor of the model highlighted the importance of feature-neutral approach to enable the neural network to detect senescence from diverse image data. Moreover, it allowed the researchers to discover age-dependent and senescence-related alternations in nuclear morphology [58]. Another CNN-based model called Deep-SeSMo achieved 93% accuracy in quantitative assessment of cellular senescence from cellular morphology images [59]. This model was used to identify compounds with senolytic properties, yielding four substances with anti-senescent and anti-inflammatory properties, one of which, terreic acid, was not previously documented to have senolytic properties.

### DL for biomarker of aging analysis: introduction to aging clocks

Aging clocks are computational models that leverage biomarkers of aging to estimate biological age [60]. The development of clinically relevant aging clocks was initiated by S. Horvath and Hannum group in 2013 with the introduction of ML-based models, which have since profoundly advanced aging research [61, 62]. These models evolved in 2020ies to integrate multiple parameters such as multi-omics data [63], enabling the assessment of organ-specific aging [64], as well as the risk of age-related diseases [65]. While ML-based aging clocks have been instrumental in shaping modern aging research, this review will focus on DL-based aging clocks, due to their potential to enhance the precision of biological age estimation.

**Figure 4:**
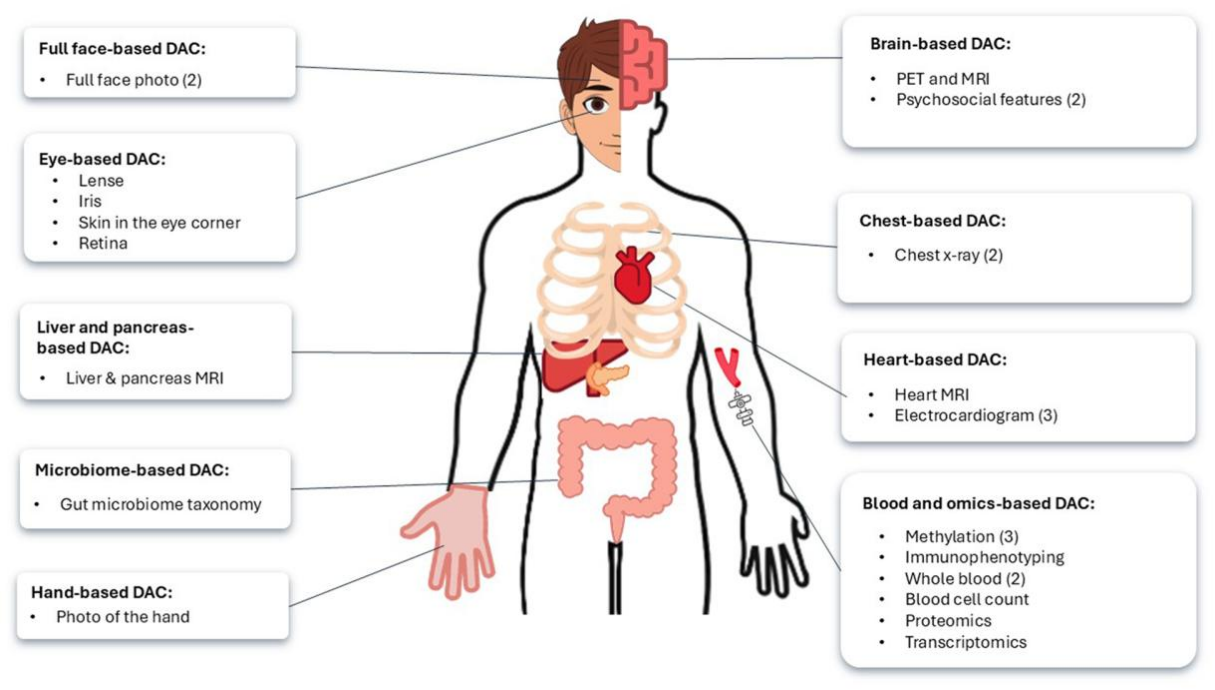
**Anatomical and biological sites assessed by deep aging clocks (DACs) to estimate biological age.** The brackets indicate the number of DACs reviewed for each category.

Table 1 compiles the list of deep aging clocks (DACs), specifying their mean absolute error (MAE) in years and type of training data. Figure 4 represents a schematic overview of DACs identified in this review, categorized by the biological or anatomical regions they assess.

**Table 1:** Deep aging clocks based on deep neural networks.

**Year**	**Authors**	**Clock name**	**Accuracy**	**Type of DL**
2016	Zhavoronkov et al.	First Deep Aging Clock	MAE = 5.5 based on blood biochemistry and sex	Ensemble of 21 feed forward DNNs with varying architectures
2018	Mamoshina et al.	Hematological age	MAE = 5.55 (entire ensemble) based on 20 blood biochemistry markers, cell counts, and sex (3 studied populations had different MAE)	Feed Forward DNN
2018	Bobrov et al	PhotoAge Clock	MAE= 1.9 based on the entire face photos	CNN
2019	Karargyris et al.	Chest age	MAE unspecified R^2 =0.89 on posteroanterior and anteroposterior Xray chest images in best validation	CNN
2019	Mamoshina et al.	Blood Biochemistry Clock	MAE = 5.78 based on 24 features including blood biochemistry	Feed Forward DNN
2019	Zhavoronkov et al. (Granted patent: US2019027289 0)	Deep Proteomic Age	MAE= 6.696 years in whole blood samples and 11.46 years for the general DNN age predictor	DNN
2020	Zhavoronkov et al. (2022)	PsychoAge	MAE = 6.7 based on psychosocial features	Feed forward DNN
2020	Zhavoronkov et al. (2022)	SubAge	MAE = 7.3 based on psychosocial features	Feed forward DNN
2020	Galkin et al.	Microbiome Age	MAE=5.9 based on microbiome taxonomic profiles (types of microbes)	Feed forward DNN
2021	Galkin et al.	DeepMAge	MAE = 3.21 in cross validation based on DNA methylation profiles	Feed forward DNN
2021	Sayed et al.	iAGE	Average reconstruction error for chronological age = 15.2 years, trained on deep immunophenotyping	Guided auto-encoder (type of DNN)
2021	Lima et al.	Heart ECG based DAC	MAE=8.38 years based on heart ECG data	CNN
2021	Raghu et al.	CRX-Age	MAE undisclosed. C-statistic of 0.751 for all-cause mortality in PLCO multivariable models.	CNN
2022	Lee et al.	Brain Age clock	MAE=3.43 based on PET and 4.20 for MRI	Feed forward CNN
2022	Nusinovici et al. (2022)	RetiAge	MAE undisclosed. Spearman’s rank correlation coefficient between chronological age and RetiAge = 0.62	CNN (visual geometry group)
2022	de Lima Camillo et al. (2022)	AltumAge	MAE= 3.563 based on CpG sites methylation from various human tissues	Feed forward DNN
2022	Le Goallec et al. (2022)	AbdAge	MAE =2.94 years trained on liver and pancreas MRI scans	CNN
2022	Chang et al. (2022)	ECGAge	MAE= 6.89 years based on electrocardiogram measurements	DNN
2022	Libiseller-Egger et al. (2022)	Heart ECG based DAC	MAE =6.1 on 12 signal electrocardiogram measurements	CNN
2023	Li et al. (2023)	LensAge	MAE=4.88 Years in diffuse-light mode at the image level, trained with the images of human lenses	CNN
2023	Lahza et al. (2023)	Iris color intensity-based aging clock	MAE=2.43 years based on eye images focused on iris color intensity	CNN
2024	Prosz et al. (2024)	XaiAge	MAE =2.83 years, trained on DNA methylation samples	DNN
2024	Zapaishchykova et al., (2024)	AgeDiffuse	MAE=1.97 in external validation on five specific MRI slices of healthy children and adolescents	Diffusion-based regression neural networks
2024	Georgievskaya et al.	Hand Age Clock	MAE=4.7 years trained on dorsal hand images	CNN
2024	Siontis et al.	Heart ECG and MRI based DAC	MAE= 6.9 based on heart MRI and ECG data	CNN

### DL in analysis of molecular and physiological biomarkers of aging

The first DAC was constructed in 2016 by A. Zhavoronkov and colleagues. The model was trained on blood biochemistry data of reasonably healthy individuals. The clock had the MAE of 5.5 years when estimating chronological age over 10 years [66, 67] (Table 1). In 2018, Mamoshina et al. constructed a hematological DAC, trained on standard laboratory tests from 3 different populations, showing the difference between their aging rates [68]. Moreover, P. Mamoshina and colleagues developed the hematological aging clock showing that smokers have a more accelerated aging rate than non-smokers [69]. Using the biochemical blood data and cell count of non-smokers, the group trained the DNN to establish the smoking status based on blood chemistry and biological sex, demonstrating the capabilities and interpretability of DNNs for advancing aging research. With the global population aging at an unprecedented rate, there is a need to extend healthy productive life span. This review examines how Deep Learning (DL) and Generative further test the efficiency, the researchers trained the DNN to establish the smoking status based on blood chemistry and biological sex, demonstrating the capabilities and interpretability of DNNs for advancing aging research.

DNNs can be used to improve epigenetic aging clock accuracy. Galkin et al. (2021) published the first deep methylation clock [70]. The model performed with MAE of 3.21 years in cross-validation. de Lima Camillo et al. (2022) constructed a multi-tissue DAC “AltumAge” to analyze the non-linear interactions among the CpG sites for age prediction [71]. Moreover, this DAC outperformed ML-based epigenetic aging clocks in terms of MAE (3.563 years), mean squared error and Pearson’s correlation coefficient. XAI-AGE, a deep epigenetic clock trained on DNA methylation data, had MAE of 2.83 years [72]. Compared to ML-based epigenetic clocks, XAI-AGE utilizes DNNs along with the attribution technique DeepLIFT, which allows for comparing and contrasting the importance of CpG sites, genes, or biological pathways to biological age prediction.

### DL in analyzing digital biomarkers of aging: domain-specific deep aging clocks

A domain-specific DAC is a predictive tool that focuses on estimating biological age based on data from a specific organ, system, or modality, rather than providing a comprehensive analysis of the entire body. The clock is often based on organ or body part-specific imaging, as visually represented in Figure 4. The performance and training details of these clocks, including MAE, are listed in Table 1. Numerous CNN- based aging clocks are trained on facial images, especially on different parts of the human eye. Lahza et al. (2023) trained CNN on eye images and videos, creating an aging clock based on iris color intensity with MAE of 2.43 years [73]. LensAge was developed by training CNNs on human eye lens pictures and achieved the MAE of 4.88 years at the image level [74]. RetiAge, another CNN-based DAC, was trained on retinal scans, and correlated well with all-cause, cardiovascular and various cancers mortality [75]; accuracy was high (Table 1), however, MAE was unspecified. In 2018, Bobrov et al. used high-resolution pictures of eye corner wrinkles to train a DNN called PhotoAgeClock to best predict chronological age, with an MAE of 1.9 years, which performs better than the CNN-based clocks of the entire face [13, 32, 76]. Georgievskaya et al. (2024) also developed a full-face aging clock using CNNs, achieving a MAE of 4.1 years [77]. Their analysis identified the corners of the mouth and the corners of the eyes as the most predictive features of age, findings that align with the low MAE reported in Bobrov’s PhotoAge model. Moreover, Georgievskaya’s team pioneered the first hand-based aging clock, leveraging CNNs to analyze dorsal hand images, which predicted biological age with an MAE of 4.7 years.

Lee et al. (2022) developed 3D-DenseNet models trained on feed-forward CNNs to predict brain age in healthy individuals [78]. The training dataset included Fluorodeoxyglucose Positron Emission Tomography (FDG PET) and Magnetic Resonance Images (MRI) brain scans of dementia patients and cognitively unimpaired controls. The clock performed with MAE of 3.14 years in predicting brain age when trained on FDG PET scans and MAE of 3.51 years when trained on MRI scans. Moreover, specific DL techniques, such as occlusion, were used to assess the clock’s sensitivity to different input regions and revealed an age-dependent saliency pattern that varied across different types of brain scans.

Cole et al. (2017) created Brain-Age DAC by training CNNs on raw gray matter MRI images of healthy individuals, achieving MAE of 4.65 years [79]. Moreover, the analysis of MRI scans of monozygotic and dizygotic twin pairs allowed for the heritability assessment of the brain’s chronological and biological age.

Another exemplary use of DL to measure brain’s chronological is “AgeDiffuse”: a diffusion dual-guidance probabilistic regression model assessing the brain age from childhood through young adulthood, focused on age-related changes in brain volume and structure on MRI scans [54]. Although this model is not a DAC, as it does not measure biological age and is rather focused on development-related than aging-related changes, its remarkable performance of MAE of 1.97 years in external validation, makes it a promising strategy for future DAC construction. Notably, it is the first open-source, implementable DL-based brain age prediction model released to the scientific community.

Heart-based DACs have also been developed. Chang et al. (2022) constructed a DAC with attention mechanisms based on electrocardiogram (ECG) scans, having the MAE of 6.89 years [80]. Lima et al. (2021) also constructed a heart ECG clock using CNNs, and indicated the positive correlation between the biological age, determined by their clock, and mortality risk [81]. Libiseller-Egger et al.’s (2022) ECG-based clock showed positive correlations between heart age and BMI, smoking status and blood-pressure [82]. Moreover, the group correlated the difference between the participants chronological age and their ECG hear age with genome-wide association study data finding 8 loci linked to the difference between chronological and heart age, suggesting that cardiovascular aging is primarily influenced by genes specific to the cardiovascular system rather than intrinsic aging mechanisms. Siontis et al. (2024) compared the heart age determined by CNN analysis of heart MRI scans and ECG data, favoring the accuracy of MRI-based heat age prediction [83]. Chest X-ray DAC constructed by Kararygris et al. (2019) offers insights into thoracic aging with high accuracy indicated by the coefficient of determination (r^2) of 0.89 [84]. Raghu et al. ’s (2021) chest X-ray DAC, CRX-Age, demonstrated a strong positive correlation with both all-cause and cardiovascular mortality [85]. An increase in CRX-Age was found to be a more robust predictor of these mortality risks compared to an equivalent increase in chronological age.

Aging rate is different across various tissues in an organism [86]. Le Goallec et al., (2022) leveraged this information and trained a DAC on liver and pancreatic MRI scans developing the first clock for abdominal aging rate [87]. This DAC achieved the MEA of 2.94 years (Table 1).

Notably, aging clocks and DAC can yield more scientific data than just the rate of aging. Many of the clocks listed in Table 1 have contributed to advancing the level of knowledge of the subdomains of aging research. “iAGE” clock development contributed to establishing CXCL9 chemokine as a major contributor to age-related chronic inflammation [88]. Other clocks evidenced the relationship between smoking and aging rate [69], established the partial heritability of the way of abdominal aging [87], or identified dorsal hand areas where skin aging is the most pronounced [77].

### DACs impact on aging clocks evolution

DACs have accelerated the development of more accurate and versatile aging clocks addressing several key challenges in the field. Firstly, DACs have improved the accuracy and reliability of age prediction models by leveraging large-scale datasets and complex patterns within the data that traditional models could not detect [66]. Traditional biomarker discovery methods often struggled with high-dimensional data and noise [89]; DACs have overcome these issues through advanced feature extraction and noise reduction techniques inherent in deep learning algorithms [66]. However, DACs are not entirely free from these issues, as discussed in the “limitations” section.

Another significant advantage of DACs is their efficiency. Traditional methods often required labor-intensive and time-consuming steps, such as manual feature selection and validation [90]. In contrast, DACs employ end-to-end learning, which automates the entire process from raw data input to final prediction, including feature selection and model training. This approach reduces the time required for biomarker discovery [19, 91].

DACs have facilitated the integration of multi-omics data, including genomics, transcriptomics, proteomics, and metabolomics, providing a comprehensive view of the aging process that was previously unattainable [69, 92]. This multi-omics approach has led to the identification of biomarkers that are robust and reflective of biological age across different biological systems. Moreover, DACs have enabled the discovery of biomarkers that are not only predictive of chronological age but also associated with age-related diseases and functional decline [19, 88, 93, 94]. This has opened new avenues for early diagnosis and personalized interventions in age-related conditions.

### Transformer models for aging research

In aging research, transformer models serve as generalized computational frameworks that emulate the behavior of biological systems across multiple contexts. Prior to addressing the topic, it is essential to clarify that this field is still in its early stages of development. Notably, three of the four referenced transformer models are available as preprints and, as of November 2024, have not been subjected to formal peer review.

Transformers, unlike traditional aging clocks developed using specific biomarkers for measuring a focused parameter of aging, can synthesize information from diverse tissues, species, and experimental conditions, incorporating multiple omics layers to enable complex simulations of biological processes and predict potential aging interventions (preprint, [45]). Built upon transformer-based architectures, these models utilize DL techniques, such as LLMs and diffusion models [95].

The first transformer model for drug and biomarker discovery and aging research, Precious3GPT (P3GPT), was developed by Galkin et al., (2024; preprint [45]). P3GPT is a multimodal, multi-omics, multi-species transformer leveraging Retrieval-Augmented Generation to maintain the most up-to-date integration of peer-reviewed literature. The model is conversational, allowing users to customize queries based on parameters such as species, cell type, pathway, gene, tissue, or gender of interest. The prompted model generates outputs, including lists of compounds with desired properties, up- and down-regulated genes, and other relevant information tailored to the specific inquiry. It is also capable of generating novel hypotheses. Furthermore, P3GPT can produce 3D molecular maps of identified compounds—ranging from approved drugs and traditional medicine extracts to novel molecules—and refine searches to highlight structurally similar compounds, facilitating novel compound discovery. In a final *in vitro* validation, P3GPT proposed 22 compounds for evaluation in a cellular senescence model, eight of which—including maslinic acid, estradiol cypionate, and dapsone—exhibited senomorphic effects without cytotoxicity.

In 2024, two foundational transformer models constructed to analyze complex DNA methylation patterns were published within 6 days from each other: MethylGPT by Ying et al. (2024, [96]; preprint) and CpGPT by de Lima Camillo et al., (2024, [97]; preprint). The key difference between MethylGPT and CpGPT lies in their focus and versatility. MethylGPT is tailored toward achieving high accuracy in specific tasks like age prediction and disease risk assessment, excelling in tissue-specific methylation analysis and resilience to missing data ([96]; preprint). Its strength lies in contextual CpG embeddings, and a robust transformer-based framework designed for aging-related research. Conversely, CpGPT is designed as a more versatile tool for multi-purpose epigenetic tasks, including zero-shot imputation, array conversion, and multi-species analysis (2024; [97] preprint). CpGPT integrates sequence, positional, and epigenetic contexts through a more adaptable transformer++ architecture, making it highly generalizable across different platforms and biological conditions. This versatility allows CpGPT to address a broader range of applications compared to MethylGPT’s task-specific focus.

Application of transformer models could lead to more efficient aging therapeutic development processes, potentially reducing the time and cost associated with bringing new treatments to market. For example, they can be used to identify new clusters of senolytics, repurpose existing drugs for aging-related conditions or identify dual-purpose targets. A transformer called BioGPT, trained on aging-related data, including genes co-mentioned with “aging” in PubMed (normalized for prevalence), targets of drugs and compounds with anti-aging properties from various databases, and PubMed abstracts focused on age-associated diseases and risk factors, was used to identify two novel dual-purpose targets: *CCR5* (linked to inflammation and AD) and *PTH* (PTH hormone serum levels elevate with age) ([48, 98, 99].

## AI-driven dual-purpose drug discovery and aging-oriented drug repurposing

Several notable companies utilize DL, GenAI, and aging research as a platform for drug discovery to target age-related diseases and the underlying biological mechanisms of aging. Calico, founded in 2012, *Insilico* Medicine, founded in 2014, BioAge Labs, founded in 2015, all have drugs in human clinical trials [100–102]. However, a few biotechnology companies, which are focused on aging, developed software accessible to others. For example, *Insilico* Medicine stands out for its development of AI-driven platforms, which can be licensed to researchers and companies for target discovery and molecule design. Academic groups such as Huang et al. (2021) [103], academic-enterprise partnerships such as Polykovskiy et al. (2020) [104] or Rozemberczki et al. (2022) [105] publish their DL libraries and benchmarking platforms in the open-source models. Nevertheless, most for-profit companies in this sector focus primarily on developing proprietary therapeutics and technologies for their own use. *Insilico* Medicine developed Pharma.AI, a comprehensive tool for AI drug discovery, allowing for novel target discovery (PandaOmics), target-specific small molecule generation (Chemistry42), as well as designing clinical trials (inClinico) [106].

### AI in drug repurposing for age-related diseases and the underlying aging process

The biochemical bases of aging and many diseases are highly interconnected; addressing them together could be more effective than treating them separately [107]. AI has allowed great acceleration in finding dual-purpose targets for aging and diseases and generating target-specific small molecules. Aging, while a natural physiological process mediated by numerous biological and genetic pathways, has not been formally recognized as a disease, so it cannot be treated as such under current medical standards. However, drugs like Metformin and Rapamycin, initially developed for other medical conditions, extend lifespan in animal models, positioning them as potential dual-purpose drugs targeting both aging and disease [108]. DL is heavily used to search for drugs to be repurposed for aging research [109]. Liu et al. (2021) created a DL-based drug repurposing framework, following randomized controlled trial design [110]. To investigate whether a drug influences a disease, researchers use real-world data, analyzing treatment timelines and patient outcomes. Temporal patterns, such as the duration of the drug use and the timing of diagnosis, are modeled using Long Short Term Memory (LSTM) networks, which excel at handling temporal dependencies and improve network’s learning capabilities by selectively remembering or forgetting information [111]. Confounding factors like age and comorbidities are adjusted with inverse probability of treatment weighting, leading to the estimation of potential efficiency, including only statistically significant results. This framework was applied in a study aiming to repurpose drugs for Alzheimer’s disease (AD) [112], however, it did not perform significantly better than the ML repurposing model, on ensuring that baseline factors are similar between groups in the simulated trials. Muniyappan et al. (2024) developed a DL framework called “DRADTiP” (Drug Repurposing for Aging through a Drug-Target Interaction Prediction) with an objective to finding human lifespan-prolonging compounds based on genetic and pathway data [113]. Their model achieved higher precision scores than Liu et al.’s (2021) framework. Huang et al. (2024) constructed a zero-shot drug repurposing foundation model called “TxGNN” based on graph neural networks [114]. This model can predict potential drug repurposing candidates without requiring prior specific training data on the exact disease-drug combination being evaluated, by identifying biochemical pathways, phenotypes and pathologies related to the disease in the prompt. In the context of aging research, Progeria syndrome, a rare genetic disorder characterized by accelerated aging and caused by mutations in the *LMNA* gene, could serve as a prompt for the model to identify drugs targeting pathways associated with the disease. These pathways may also provide insights into the fundamental mechanisms of aging, as Progeria shares key biological features with normal aging processes, such as nuclear instability, cellular senescence, and DNA damage [115].

### AI for discovering aging-related targets

AI application led to the discovery of multiple dual-purpose targets for aging and disease. Pun et al. (2023) screened 16 740 healthy samples and 19 334 protein-encoding genes yielding 51 known and 23 novel dual-purpose targets for aging and various types of cancer [116]. Among them, the researchers emphasized the therapeutic potential of targeting *KDM1A* gene, an essential regulator of autophagy in humans and life extension in *C. elegans* after knockdown. Other AI-discovered dual-purpose targets include *CNGA3, GLUD1*, and *SIRT1* for aging and glioblastoma multiforme, and *APLNR* and *IL23R* for aging and multiple age-related diseases [44, 117]. Moreover, Pun et al. (2022) discovered 28 targets for Amyotrophic lateral sclerosis, which is an age-related disease [118]. Novel targets for AD were found using DL and a computational tool that predicts the propensity of protein regions to undergo liquid-liquid phase separation [119].

### AI for small molecule generation for aging-related targets

GenAI techniques such as autoencoders, GANs, flow-based approaches, evolutionary algorithms, or LLMs are used to generate novel small molecules with target-specific properties [106]. The main molecular representations are string-based, graph-based, and 3D-based. By the very nature of generative models, the algorithm learns and improves the molecule design until it reaches the most desirable form [120]. An AI generated small molecule against an AI-discovered target for idiopathic pulmonary fibrosis, an age-related lung disease, recently completed the phase 2a clinical trial [121]. The drug (INS018_055) showed anti-inflammatory and anti-fibrotic effects in multiple *in vivo* and *in vitro* experiments, as well as almost 100 ml improvement in forced vital capacity compared to placebo [102, 122]. Moreover, in the hallmarks of aging assessment, INS018_055 scored highly against six hallmarks of aging, including stem cell exhaustion, altered intracellular communication, and extracellular matrix stiffness making it a potential dual-purpose drug candidate [122].

### AI in clinical trial improvement: accelerating bringing the longevity therapeutics to the market

All the AI-generated therapeutics must undergo the same clinical validation as conventionally developed ones. Regardless of the development method, getting a drug to clinical trials is expensive, and is a significant backlash to longevity biotechnology companies, if the program fails. AI can help design, fine-tune, and assess the probability of success in clinical trials by e.g. identifying eligible participants through electronic health records or using GenAI to convert protocols into detailed procedures [123, 124]. One of the models uses a multimodal approach in ML to integrate a variety of data, including multiomics data, drug structure, preclinical data, as well as related publications, grants, patents, and trial protocols, reaching 79% accuracy in the trial outcome prediction [125]. If a company strategically limits investment in potentially ineffective clinical trials by prioritizing compounds with a relatively high probability of success based on model predictions, it can reallocate resources to optimize these compounds or develop new ones, thereby potentially accelerating the timeline for delivering effective treatments to market. Since multiple gerotherapeutics are in clinical trials as of 2024 [126], there is an opportunity for applying AI to increase efficiency of clinical data analysis and preparation of future trails, potentially indicating faster approvals, if the compounds prove effective.

### AI in geroprotector discovery

Geroprotectors are compounds that aim to slow down the aging process or protect against age-related decline [127]. Using computational analysis of gene expression data can significantly accelerate their development compared to direct biochemical or physiological experiments [128]. In 2016, Aliper et al. developed GeroScope, a method utilizing DNNs and signaling pathway scores, to identify potential geroprotectors [129]. The system identified compounds that mimic youthful expression patterns within aging-related pathways by analyzing gene expression profiles from young and old subjects. The compounds shortlisted by the algorithm were validated *in vitro* using senescent human fibroblast cultures. Compounds such as PD-98059, a MEK1 inhibitor, were shown to act as geroprotectors and rejuvenating agents [129]. In 2017, Aliper et al. trained a DNN with transcriptome response data using gene expression profiles and signaling pathway scores to efficiently screen a vast number of natural compounds for natural alternatives to Metformin and Rapamycin [128]. The algorithm yielded 871 natural compounds, 2 of which, geldanamycin and withaferin A, were proposed to best mimic Metformin and Rapamycin's anti-aging and anti-cancer effects without their adverse effects.

Wong et al. (2023) constructed a graph neural network to discover small molecule senolytics [130]. The group screened drugs approved by US Food and Drug Administration, as well as senolytic drugs in clinical trials, and experimentally validated the drugs that the network output as having senolytic properties. They later constructed another DNN predicting senolytic activity from a compound’s chemical structure and validated it on the Broad Institute Library of compounds, eventually yielding 3 compounds with senolytic properties. The authors tested one of the compounds in mice, revealing that it decreased senescent cell burden and senescence-associated mi-RNA expression.

While AI has shown promise in identifying potential geroprotectors, it also plays a crucial role in addressing challenges associated with drug interactions. Community-dwelling adults are increasingly exposed to polypharmacy, defined as the regular intake of five or more medications, which often leads to cross-reactions that can reduce drug effectiveness or amplify adverse effects [131]. In the longevity community, the widespread popularity of supplements raises similar concerns, as their excessive or unregulated use can lead to cross-reactions and cumulative toxicities. Decagon is a tool that uses graph convolutional neural networks to analyze multimodal graphs, integrating protein-protein and drug-protein interactions [132]. It predicts the occurrence of a side effect from a drug combination and identifies the specific type of side effect.

As the number of identified geroprotectors continued to grow, the need for a comprehensive database became increasingly evident. In 2015, the first geroprotector database “geroprotectors.org” was established, featuring 250 experiments covering over 200 known or candidate geroprotectors [133]. In March 2024, this database contained 2408 references for geroprotective compounds. DrugAge is a manually curated database of geroprotectors that as of March 2024, contained 2296 lifespan-elongating assays in 1097 distinct drugs in 37 distinct species [134].

## AI-enabled healthy longevity medicine

Healthy longevity medicine is a multidisciplinary discipline that aims to optimize health and extend healthspan through targeting the underlying causes as well as early sign of aging process [135]. The field is extensively health data-driven, creating an opportunity for applying AI to improve measurement accuracy, as well as facilitate the interpretation of results [36]. Moreover, such data gathering will further facilitate AI-based aging research, which heavily relies on longitudinal biochemical data availability [107, 136].

### AI in disease diagnosis

Along with prevention, early diagnosis of age-related diseases such as cardiovascular disease, type 2 diabetes mellitus and age-related functional decline is central to healthy longevity medicine. AI has enabled unprecedented precision in analyzing clinical data related to these conditions, increasing the diagnostic accuracy, defined as the correctness of diagnosis [137, 138].

By the time of writing this review, there are no drugs that cure AD, but there are some that delay the symptoms and improve patients’ life quality [139]. However, early diagnosis is necessary for these drugs to be effective. A systematic review by Arya et al. (2023) demonstrated that diagnostic methods for AD based on CNNs can achieve a diagnostic accuracy of 98.6% [138]. In comparison, RNN allows for 91.2% accuracy, marking a significant improvement from 85.71% achieved with ML standard vector machine classifiers. An algorithm combining CNN and RNN for longitudinal and spatial data analysis from MRI scans in DL-based AD diagnosis achieved 91.3% diagnostic accuracy [140]. Qiao et al. (2018) created an automatic AD diagnostic tool by utilizing Directed Acyclic Graph neural network [141]. The group trained the tool on fMRI images via multivariate data-driven feature extraction and achieved 95.59% accuracy. These examples show that DL allows for accurate and early diagnosis of AD, facilitating timely interventions. However, disease severity assessment is also important in designing the best action plan for the patient. A DL tool based on resting-state fMRI data and a Three-Dimensional CNN achieved an accuracy of 92.3% in assessing AD’s severity [142].

Beyond AD, AI has shown promise in diagnosing and assessing various other age-related conditions. Changes in the gut microbiome are a proposed biomarker of aging serving as a base for building aging clocks and diagnostic tools for risk factors such as obesity or cardiovascular diseases [143–145].

In the field of cardiology, Hannun et al. (2019) trained CNNs to process raw, single-lead electrocardiogram signals to extract meaningful features, enabling the model to learn and recognize complex patterns associated with different cardiac conditions, yielding 12 distinct cardiac arrhythmias [146]. This DL model was evaluated against the consensus of expert cardiologist diagnoses. It achieved high Area Under the Curve scores for all rhythm classes, showcasing its ability to accurately distinguish between various cardiac abnormalities.

### AI in genetic risk prediction for healthy longevity medicine interventions

Healthy longevity medicine emphasizes not only the early diagnosis of diseases but also the comprehensive assessment and proactive mitigation of associated risks. DeepPRS is a DL tool used to assess individuals’ polygenic risk scores of AD, inflammatory bowel disease, type 2 diabetes mellitus, and breast cancer with genome-wide genotype data [147]. The study demonstrates the effectiveness of AI in enhancing genetic risk prediction models, showing that DeepPRS can identify individuals at higher risk for these diseases based on genotype information available at birth. Such tools aid longevity physicians in making informed clinical decisions.

Beyond analyzing existing genetic data, researchers are developing novel methods to represent genetic information in ways that are more amenable to AI analysis. In 2024, gene vectorization, inspired by the word2vec model from natural language processing was applied to transform genes into numerical vectors [148]. By embedding genes within a high-dimensional space, where the position of each gene vector represents its biological functions, as identified in databases like Gene Ontology and ARCHS4, these gene vectors, known as FRoGS (Functional Representation of Genes in Space), incorporate both known and empirical gene functions, enabling a comprehensive representation of each gene’s role in various biological processes. By starting with pre-trained gene functional embeddings, the researchers can fine-tune their models on smaller datasets specific to aging, enhancing model performance and reducing the need for extensive new data.

### AI in continuous health monitoring: application to healthy longevity medicine

Healthy longevity medicine is heavily dependent on monitoring data about a patient’s health. The comprehensive set of data gathered from all diagnostic tests is the base for longevity physicians to prescribe longevity interventions, informs them about the success of interventions, and can be used to train AI models, which can be further used to advance aging research [136]. Continuous health data monitoring often involves sequential data that benefits from both spatial and temporal analysis. The hybrid approach combining CNN and LSTM allows the models to achieve high accuracy of 94.71% in blood glucose monitoring [149] and the sleep-wake detection superior performance to traditional models [150].

While continuous monitoring provides real-time data on an individual’s health, researchers are also exploring ways to simulate and predict health outcomes using advanced AI models. This has led to the development of digital twins in healthcare: virtual models that replicate the physical and biological processes of the human body developed using ML [151]. These models allow researchers and clinicians to simulate and analyze the effects of interventions, lifestyle changes, or drug treatments on an individual’s aging process without direct experimentation. Unlearn.AI is developing digital twin models for AD [152]. PreComb developed a 3DTwin® Digital platform: an ML-based data analysis tool that uses data mining techniques for testing the patient-specific effectiveness of targeted cancer therapies, even without target identification [153].

## AI for lifespan psychology research

Generative and predictive AI systems can be trained on behavioral data to assist with psychological aging research. Socioemotional selectivity theory states that the longer people estimate to be alive, the more they prioritize activities with long-term benefits, such as gaining knowledge or expanding their social network [154]. Consequently, when they feel that they will die relatively soon, they choose immediately emotionally gratifying activities. This follows long-term oriented lifestyle choices, which in turn improve healthspan expectations [155]. Mental and physical health are highly interdependent, which suggests the same for biological and psychological age.

Recent studies have begun to explore these connections between psychological factors and biological aging using AI-driven approaches. In 2022, a study published by A. Zhavoronkov’s group showed the correlation between subjective well-being and biological age as estimated by a feed-forward DNN predictor [156]. Psychological factors commonly seen as “negative,” such as loneliness, anxiety, or lack of focus, cumulatively increased the biological age (calculated by blood biomarker analysis) by 1.65 years. Further analysis demonstrated the most important features implicated in both biological and psychological age; “being married” seems to be associated with a slower pace of aging compared to non-married individuals. In this study, “living in rural areas” and “rarely feeling happy” seem to accelerate the biological age the most [156].

## Limitations and challenges of using AI in aging research

Although AI has shown great potential in advancing aging research and healthy longevity medicine, addressing several key limitations is essential for its ethical and effective application. Aging clocks often exhibit inherent error rates and biases. For example, older individuals may be predicted as younger or vice versa, depending on cohort characteristics, as some predictions are statistically driven rather than purely biologically informed [75].

Othmani et al. (2020) highlighted limitations in CNN-based frameworks for automatic age estimation from facial images, showing that variations in facial expressions, lighting, and occlusions can adversely affect accuracy [157]. Additionally, underrepresented ethnic groups, particularly Black individuals, experience reduced performance due to a lack of generalization; increasing training dataset diversity could mitigate this issue. These biases highlight the importance of ensuring diverse and representative datasets for robust AI models. Several studies found that ML algorithms estimated the chronological age of full-face photos of older adults and females less accurately than the photos of younger and male faces respectively [158, 159]. Ganel et al. (2023) argue that such age misestimation may result from a stronger regression to the mean effect, where estimates tend to shift toward the average age of the training data [158]. Moreover, they partially attribute the age misestimation to the lack of photos of individuals over 70 in the sample, which points at the importance of diverse training data while constructing CNN based aging clocks. Georgievskaya et al. (2023) analyzed the sources of AI bias and categorized such underrepresentation of a specific parameter in the data as negative set bias [160]. The lack of variety in data can be addressed via GANs or other GenAI techniques able to create synthetic data. Confounding bias is equally important to consider while constructing aging clocks. It occurs when additional factors distort the relationship between the primary variable and the outcome, leading to inaccurate results [160]. In AI models applied to aging, this might happen when using skin elasticity to estimate biological age but failing to account for lifestyle factors like smoking, which also affect skin elasticity. Without including smoking data, the model may incorrectly attribute changes in skin elasticity solely to aging, resulting in biased and misleading predictions. Regarding the CNN clocks using full face images, the quality of the photo and its attributes such as brightness or occlusion is especially important to the accuracy of age prediction [160].

AI applications in biomedicine, face challenges related to the interpretability and transparency of predictions [161, 162]. The “lethal prejudice” occurs in situations where biases embedded in AI systems lead to life-threatening consequences or disproportionately harm certain groups [163], such as mentioned before inaccurate prediction of biological age for older adults in certain ethnic groups due to underrepresentation in training datasets. Haibe-Kains et al. (2020) caution against prioritizing accuracy of the models over their interpretability in medical applications, arguing that in the current medical system, human physicians have the final saying in decision making; over-reliance on “blackbox”, unexplainable AI may lead to doctors not analyzing the patient’s situation carefully enough, potentially leading to harm [161]. Moreover, such cases introduce the challenge of distribution moral and legal responsibility for the harm.

Explainability can be mitigated by employing a second AI model, which would explain the actions of the first [14]. However, Haibe-Kains et al. (2020) argument that this introduces another level of complexity and relies on incomplete information extraction [161].

The creation of digital twins, which require extensive, high-quality datasets, is similarly hampered by disparities in data availability across populations. Ethical issues related to digital twins in medicine are centered on the control of data. Although digital twins still remain at the organ level, the debate arises whether the company creating the twin should have rights to manipulate it without the knowledge of the “template” human [164, 165].

While AI can accelerate the discovery of therapeutic targets and compounds, the generated compounds, just like conventionally developed therapeutics, must be validated through experimental methods and clinical trials to ensure safety and efficacy.

Creating advanced DL and GenAI architectures is an expensive endeavor due to their high computational demands. Training these models often requires significant investments in infrastructure, power, and computational resources. Although the cost of graphic processing units (often used for DNN training on massive data sets, such as multi-omics DACs), has declined substantially in recent years [166], the overall expenses in creating robust AI models may remain prohibitive in resource-constrained environment, necessitating the researchers to trade cost for time-efficiency (preprint, [167].

## Future considerations

Recent attention in biopharma and aging research has increasingly focused on the potential future applications of Quantum Computing (QC). While practical implementation remains many years away [168], the eventual arrival of QC could significantly enhance AI capabilities by expediting the training process and enabling algorithms to predict more complex functions due to increased expressiveness [169]. QC is based on quantum bits (qubits) that exist in many states simultaneously (superposition), possibly allowing multiple computational paths to run simultaneously. For aging research, this could mean faster analysis of biological data, quicker identification of aging biomarkers, and accelerated drug discovery processes. This could be particularly valuable in personalized medicine and understanding individual aging processes.

In February 2024, the first quantum-classical algorithm developed experimental hit for a small molecule drug targeting KRAS was developed [170]. Three million samples (one for classical samples via LSTM, one for 16 qubit quantum samples, and one for simulated samples) were analyzed, yielding 15 novel compounds. Two of the compounds were validated as viable complex drug targets.

Aging research involves numerous optimization problems, such as molecular structure prediction, identification of optimal therapeutic targets, and optimization of intervention strategies. Quantum algorithms, like the Quantum Approximate Optimization Algorithm, can navigate these complex optimization landscapes more effectively, potentially identifying solutions that classical algorithms might miss or take significantly longer to discover [171].

Another promising future direction, already beginning to see application, is the development of autonomous AI agents. These systems combine chat-optimized LLM and ML algorithms to autonomously generate biomedical discoveries [172]. A group at Harvard University, led by M. Zitnik, has described “level 3 autonomous agents” capable of contextualizing, rather than merely identifying, biomedical discoveries. At this level, human scientists’ role is primarily to set the initial hypothesis, which the agent then verifies by proposing and executing appropriate experiments. Recent studies exemplify progress towards this level of automation. For instance, researchers at Massachusetts Institute of Technology have developed a conversational, multi-agent system for autonomous protein design, which demonstrates the ability to comprehend complex tasks, identify potential weaknesses, and develop strategies to address these issues, ultimately improving the generated outcomes [173].

Two companies, *Insilico* Medicine and Sakana AI, announced that they managed to genuinely invent in the automated way using multi-agent systems. Sakana AI’s “AI Scientist” and *Insilico’s* P3GPT, described in the “Transformers for Aging Research” section both aim to automate aspects of scientific research, but they differ significantly in their design and application scope. Sakana’s AI Scientist is built for end-to-end automation of the research process, covering idea generation, experiment planning, execution, result analysis, and even manuscript writing and peer review [174]. It is designed to autonomously run multiple iterations of research, using previous outputs to improve future experiments, enabling a continuous cycle of scientific discovery. However, its current application is primarily in AI-related fields and has limitations in handling multimodal data and visual processing, making it prone to logical and numerical errors.

In contrast P3GPT, while also leveraging AI for automation, is more specialized for biomedical research. The core of the approach involved integrating P3GPT into an autonomous system of specialized agents, each dedicated to specific components of the research workflow, such as hypothesis generation, multi-omics data integration, and compound screening (preprint, [45]).
